# Middle Ear Teratoma: Clinical and Imaging Features

**DOI:** 10.2174/1573405619666230117140658

**Published:** 2023-05-31

**Authors:** Jun-hua Liu, Wen-hu Huang, Yin Liu, Fang Zhang, Yan Sha

**Affiliations:** 1 Department of Radiology, Eye and ENT Hospital, Fudan University, No. 83 Fenyang Road, Xuhui District, Shanghai 200031, China;; 2 Department of Otolaryngology, Eye and ENT, Hospital of Fudan University, No. 83 Fenyang Road, Xuhui District, Shanghai 200031, China

**Keywords:** Teratoma, middle ear, eustachian tube, tomography, X-ray computed, magnetic resonance imaging

## Abstract

**Background:**

Teratoma is a true neoplasm composed of a number of different types of tissue derived from the three germinal layers but rarely occurs in the middle ear (ME). The features of middle ear teratomas (MET) have not been well described.

**Objective:**

The objective of this study is to explore the clinical and imaging features of MET, and report 2 rare cases of MET with ear malformation that have never been reported.

**Materials and Methods:**

The clinical, CT and MRI data of 8 patients with a pathological diagnosis of MET were collected and retrospectively mined, and 14 patients with MET reported in previous literature were also reviewed.

**Results:**

① Female, left ear predominance in MET, and the most common symptoms were otorrhea and hearing loss. ② On CT and MRI, the MET presented as an irregular soft tissue mass that was heterogeneous, with fatty tissue and involved multiple sites, and the ET and tympanum were correspondingly expanded and locally destroyed. ③ Mictotia with MET in two patients was presented, which was the first report.

**Conclusion:**

MET has female sex and left ear predominance. CT and MRI can be used to diagnose MET and display its extent and its relationship to the carotid canal in detail. Complete surgical excision is the definitive treatment.

## 1 INTRODUCTION

Teratoma is a true neoplasm composed of a number of different types of tissue derived from the three germinal layers, endoderm, mesoderm and ectoderm. The incidence of teratomas is 1:4000 births [1]. Teratomas can appear in any part of the body; they usually appear in midline and paraaxial locations, with the sacrococcygeal area being the most common site, followed by gonadal, mediastinal, head and neck, and retroperitoneal regions. Other rare locations have also been reported [2].

Teratomas of the head and neck account for 5 to 15% of all teratomas and predominantly involve the nasopharynx and neck [3, 4]; presentation in the middle ear is extremely rare. Teratomas located at different sites have a very high diversity of histological and clinical behaviours [2]. Although the features of teratomas in some sites, such as the sacrococcygeal area, have been well described, information regarding the features of MET is very limited and limited to a few case reports due to its rarity. Previous studies sporadically mentioned the clinical and imaging findings. In this paper, we present the largest number of cases of MET and review the literature to discuss its clinical and imaging features.

## 2 MATERIALS AND METHODS

### 2.1 Patients

This retrospective study was approved by the ethical committee of the Affiliated Eye and ENT Hospital of Fudan University, and patient consent was waived. We searched the key word “teratoma” in the pathological reporting system database for entries between January 2007 and November 2020. Cases involving ME, including those of the EAC, tympanum, mastoid, and ET, were selected. Diagnoses of hairy polyps or dermoids were excluded. Then, patients with MET were searched in our image reporting system database, and those with preoperative CT or MR images were included in this study.

The electronic medical records were reviewed, and patient characteristics, including sex, age, left or right side, symptoms, and especially surgical records, were collected.

### 2.2 CT and MRI Protocol

Scanning ranged from the top of the petrous bone to the tonsil. High-resolution CT of the temporal bone in our hospital was implemented with SOMATOM Definition Edge (Siemens Medical System, Forchheim, Germany). The scanning parameters were as follows: tube voltage, 120 kV; tube current, CARE Dose 4D; collimator width, 6*0.75 mm; slice thickness, 2 mm; and spacing, 2 mm. Bony algorithms and soft tissue algorithms were used for reconstruction. Particularly in paediatric patients, CT scans can deliver a significant radiation dose, increasing radiation-induced cancer risk, cognitive dysfunction due to brain damage, and lens and haematopoietic system damage. During CT scanning, we strictly followed ALARA principles to minimize the influence of radiation exposure of CT on children. Patients wore a lead cap, lead patch, lead collar, and lead clothes to cover the head, eyes, neck, chest, abdomen, and limbs.

Patients who had 0.75 mm thin images had ossicular chain multiplanar reformation (MPR) of the malleus, incus and stapes. The oblique planes were defined to optimally depict the three structures according to rotation of the reference line on axial, coronal and sagittal images.

MRI scans were performed using a 3.0-T MRI scanner (MAGNETOM Prisma, Siemens Healthcare, Erlangen, Germany) with the following parameters: thickness, 2 mm; slice gap, 0.2 mm; matrix, 512x512; axial SE T1-weighted imaging (T_1_WI) sequence (TR, 952 milliseconds; TE, 12 milliseconds); axial and coronal fat-suppressed fast spin‒echo T_2_-weighted imaging (T_2_WI) sequence (TR, 5000 milliseconds; TE, 82 milliseconds); diffusion-weighted imaging (DWI) (readout-segmented echo-planar imaging, TR, 4700 milliseconds, TE 66 milliseconds; b values, 0, 1000 s/mm^2^; thickness, 4 mm). Axial and coronal fat-suppressed T_1_WI scans were also obtained after an injection of gadopentetate dimeglumine (Gd-DTPA, Magnevist, Bayer Schering).

### 2.3 Imaging Analysis

All CT and MR images were systematically examined by two radiologists working in consensus, with 15 and 35 years of experience in head and neck imaging diagnosis. The following details of the teratomas were evaluated: morphology, density or signal, involved location, integrity of the ossicular chain (malleus, incus, stapes), and details of the internal carotid canal.

### 2.4 Literature Review

We used PubMed to search the literature using the key words teratoma, middle ear, and ET. After excluding studies that described dermoids or hairy polyps, 14 full papers written in English that described MET from three germinal layers were included.

## 3 RESULTS

### 3.1 Clinical Findings

Eight patients with MET were recruited, and 14 full papers written in English that described teratomas in the middle ear were included in this study. Finally, 22 patients were described, including 19 females and 3 males, and 16 left ears and 6 right ears were affected. The age at the time of admission ranged from one week to 48 years. The clinical features are summarized in Table **1**.

Fifteen patients had recurrent otorrhea, 2 patients had chronic otitis media that had been refractory to standard medical treatment, and the first presenting ages within three years old ranged from newborn to 1 year in 15 patients. Fifteen patients had hearing loss on the affected side, and 7 patients were not mentioned in the papers. Three patients had facial paralysis on the affected side. Clinical examination revealed an amount of pus and granulation tissue in the affected EAC in 10 patients, and a mass in the EAC was present in 8 patients after aspiration of pus. A mass in the mouth was present in one patient, and a mass in the later cervical region was present in one patient. Four patients underwent biopsy before surgery. Intraoperatively, a tympanum cholesteatoma was also found in three patients.

In our cases, seven patients underwent surgery to completely resect the mass. The final patient (No. 8) only underwent the biopsy. Microscopically, the tumours were covered with skin, which was stratified by squamous epithelium and a slightly ciliated columnar epithelium. It also contained skin appendages: sebaceous glands, sweat glands and hair follicle tissue. Well-differentiated fibroadipose tissue, cartilage, bone, muscle, nerve and salivary gland tissues were demonstrated.

Follow-up after surgery, all patients with otorrhea were well, with complete recovery from otorrhea, and all patients had no MET recurrence.

### 3.2 Imaging Findings

**Fig. (1):**
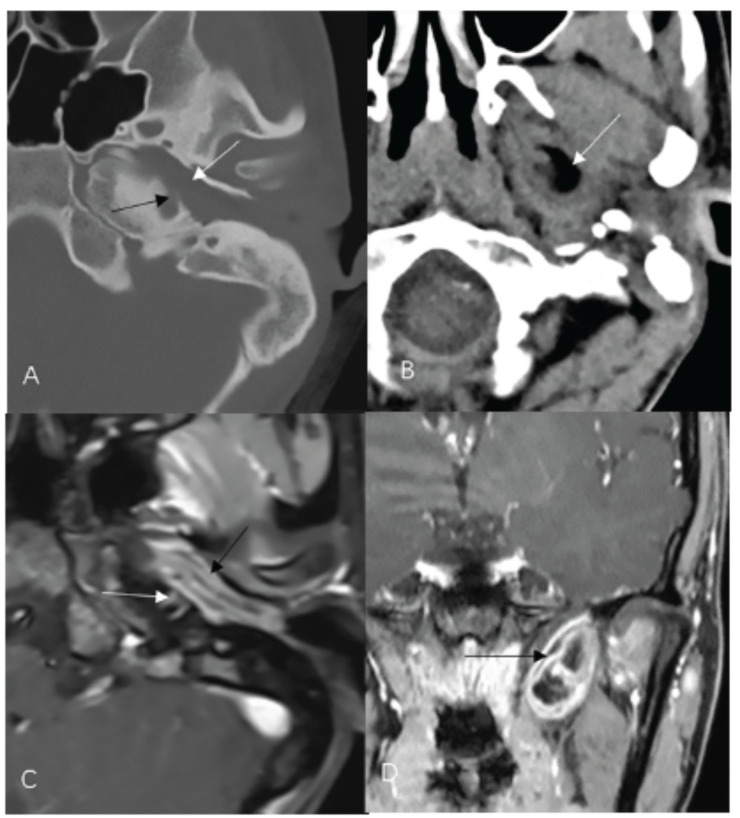
MET involved EAC, tympanum, ET and parapharyngeal spaces, patient No. 3. (**A**), axial CT Bony algorithms reconstruction imaging, internal carotid artery canal slightly destruction (black arrow), ET expanded (white arrow). (**B**), axial CT soft tissue algorithms reconstruction imaging, MET involved parapharyngeal space (white arrow), with thick-walled capsules. (**C**), axial fat-suppressed T1WI enhancement. Capsules had two layers of soft tissue sandwiched by a thin layer of adipose tissue (black arrow), internal carotid artery (white arrow). (**D**), coronal fat-suppressed T1WI enhancement. Part of the MET in the parapharyngeal space, thick-walled capsules with sandwich sign (black arrow), and central soft and capsule enhanced.

All patients had radiological examinations, including CT scans (n=20), MRI scans (n=17) and 3 patients with DWI, and one patient had X-ray films of the mastoid bone. The imaging features are summarized in Table **2**. On CT and MRI imaging, MET presented as an irregular, encapsulated, heterogeneous, soft-tissue mass. Fat attenuation or signs were present in 16 patients, teeth were present in two patients, calcification was present in two patients, and solid-cystic mixed masses were present in three patients. There was no restricted diffusion on the DWI image in 3 patients who had a DWI scan. In our cases, fat attenuation or signs were observed for all patients. On MRI, four patients displayed smooth, thick-walled, layered capsules, and the capsules had two layers of soft tissue sandwiched by a thin layer of adipose tissue in four patients, similar to a sandwich, especially on the fat-suppressed enhancement T_1_WI sequence (Fig. **1**).

MET involved multiple sites, including the tympanum, mastoid, ET, EAC, infratemporal, nasopharynx, parapharyngeal space, lateral cervical space, *etc*., and the most involved site was the tympanum (n=18), followed by ET (n=17), EAC (n=12), parapharyngeal space (n=5). The corresponding tympanum, ET, EAC expanded, anteroinferior wall or floor of the tympanum was destruction in our cases (n=6), and without bone hyperplasia and sclerosis at the edge of the bony destruction.Some important structures were involved, including the internal carotid artery canal (n=2), petrous apex and jugular bulb (n=1).

In our cases, six patients had slight destruction of ossicular chain on CT imagings, which was consistent with the surgical findings. All patients had otitis media with intact scutum.

**Fig. (2):**
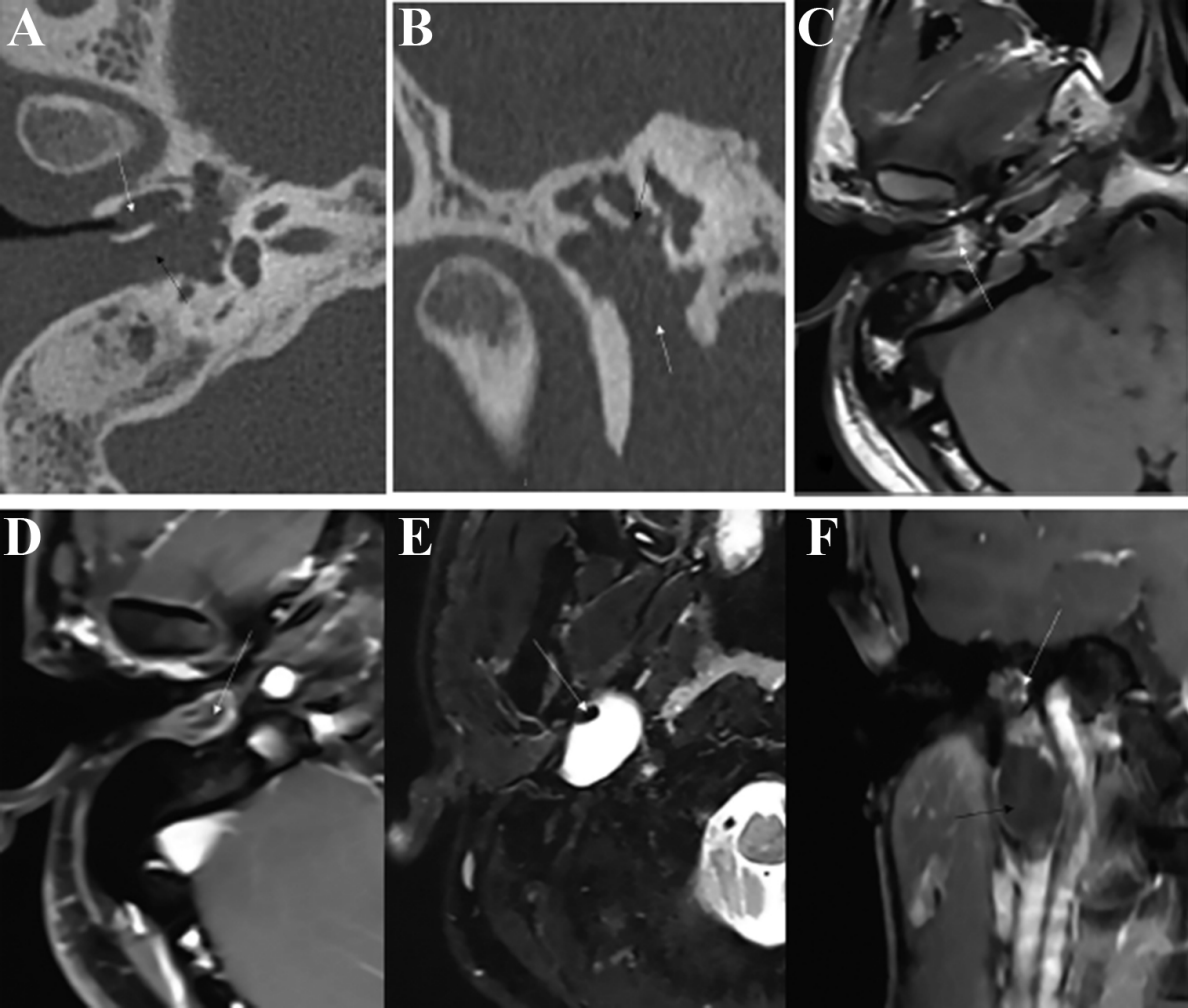
Ear malformation with MET, patient No. 8. Teratoma involved the tympanum, parapharyngeal space, and space between the posterior wall of the EAC and the mastoid, which had the appearance of two parallel bony canals. (**A**), axial CT imaging, stenosis of the EAC with soft tissue (white arrow), teratoma involving the space between the posterior wall of the EAC and the mastoid (black arrow). (**B**), Coronal MPR of the long process of the incus (black arrow); the long process of the incus was partly eroded, and the inferior wall of the tympanum (white arrow) was expanded and destroyed. (**C**), Axial T1WI imaging. MET demonstrated heterogeneous hyperintensity with little isointensity. (**D**), Axial fat-suppressed T1WI enhancement. MET showed moderate heterogeneous enhancement, and the fat tissue showed hypointensity without enhancement (white arrow) and a sandwich sign. (**E**), Axial fat-suppressed T2WI: Part of the MET in the parapharyngeal space was a cyst with a fat nodule (white arrow). (**F**), coronal fat-suppressed T1WI enhancement. Part of the MET in the tympanum showed moderate heterogeneous enhancement (white arrow), and part of the MET in the parapharyngeal space (cyst) showed no enhancement (black arrow).

In our cases, 2 patients had microtia. They both had Grade I microtia and EAC stenosis. On CT imaging, the posterior wall of the bony external auditory canal was nearly parallel to the mastoid cortex, forming a false bony canal. The teratoma involved both the meso-hypotympanum and EAC. In patient No. 7, the teratoma expanded into the posterior styloid space, and destruction of the floor of the tympanic cavity was observed. In patient No. 8, the teratoma expanded down the anterior styloid and parapharyngeal spaces, and destruction of the anteroinferior bony wall of the tympanic cavity was observed (Fig. **2**).

Preoperatively, 6 out of 22 patients were diagnosed with cholesteatoma by CT and/or MRI, and 2 patients were diagnosed with otitis media by CT.

## 4 DISCUSSION

Teratoma family includes a wide variety of lesions, and many classifications of teratomas have been proposed. Arnold [17] classified teratomas into four categories: dermoid, teratoma, teratoid tumour, and epignathi. Dermoid, also called hairy polyp, is composed of two germinal layers, the ectoderm and mesoderm, without the endoderm. Teratoma is a true neoplasm composed of a number of different types of tissue derived from the three germinal layers, endoderm, mesoderm and ectoderm. Teratoid tumours are composed of three germinal layers, poorly differentiated, immature tissue derivatives, and aggressive clinical behaviours. Epignathi is composed of three germinal layers that are highly differentiated and commonly originate from the oropharyngeal region, such as the upper jaw, palate, and sphenoid bone. In some classification [4], teratoma includes epidermoid cyst, containing derivatives of a single germ layer, usually ectoderm. Some cases of epidermoid cyst, dermoid or hairy polyps of middle ear were reported as MET [4]. However, their imaging features are different. In this study, MET is derived from the three germinal layers.

Middle ear includes tympanum, tympanic antrum, mastoid and ET, therefore the teratoma derived from the ET and extended down to the nasopharynx in one patient was included [9]. In this study, 19 females: 3 males, approximating the generally accepted sex ratio of 6 females:1 male for teratomas. 17 left ears and 5 right ears were affected, indicating an absolute predominance for female and left ear involvement. The most common presenting symptoms of MET were otorrhea and hearing loss, which is consistent with the reports [4]. Otorrhea and refractoriness to antibiotics were followed by the inability of the middle ear secreta to drain because of the obstruction and destruction of the ET. Three patients did not have otorrhea because the Ets were intact. 19 patients presented symptoms within 3 years old, including 17 patients within 1 year old; histologically, all tumours were mature, without malignant elements, and all patients had no MET recurrence. Therefore, MET could be considered a congenital benign tumour.

The less frequent symptoms included facial paralysis, local soft mass, and microtia, which was different from the report [4]. Microtia with MET has not been reported upon in the literature. Two patients in our cases had special malformations, and they both had a false bony canal behind the bony EAC. This kind of anomaly might be a syndrome that involves the first and second arches and the first cleft.

The symptoms of MET are not specific, and MET is difficult to detect by clinical examination because of the amount of pus and granulation tissue in the affected EAC. CT and MRI play an important role in the diagnosis and treatment of MET. On imagings, MET presented as an irregular, encapsulated, heterogeneous, soft-tissue mass, presenting fat attenuation or signs, teeth, calcifications, and cysts, similar to most teratomas. MET demonstrated expansive growth, involved multiple sites, including the ET, EAC and tympanum, parapharyngeal space, *etc*., and with a tendency to spread itself towards less resistant areas. The tympanum, ET, and EAC were correspondingly expanded and locally destroyed, and without bone hyperplasia and sclerosis at the edge of the bony destruction. Like most of teratoma, fat attenuation or signs were the key sign for diagnosis. MRI is superior to CT in demonstrating thin layers of adipose tissue in the capsule.

MET is extremely rare and usually accompanied with otitis media, and it is easily misdiagnosed as otitis media or cholesteatoma in the initial diagnosis, regardless of the clinical or imaging findings. In this study, 8 patients were misdiagnosed with cholesteatoma or otitis media by imagings, four patients underwent two operations to completely excise the teratoma, and 4 patients underwent an unnecessary biopsy because of misdiagnosis. The main reason for the misdiagnosis was missed or ignored teratoma in the ET or parapharyngeal space, especially on CT imagings. It is necessary to focus on the relationship between the carotid artery canal, jugular bulb and the mass, specially carotid canal, as the carotid canal may be thinned, even destroyed and remodelled by the teratoma.

Cholesteatoma is a common disease of the middle ear and mastoid, and it shares recurrent otorrhea, tympanum, EAC widening The CT features of cholesteatoma include a homogeneous soft tissue mass without fat, scutum erosion, erosion of the ossicular chain, fallopian canal, labyrinthine, Bone hyperplasia and sclerosis at the edge of the destruction cavity,*etc*. [18], while the ET, parapharyngeal space, and laterocervical space are intact. and the ossicular chain destruction was more severe than MET. Regarding teratomas accompanied by cholesteatoma, DW-MRI sequences may be a key imaging technique [16].

The differential diagnosis should include fat-containing lesions, such as lipomas, hamartomas, and other masses of the teratoma families. Lipoma is a common benign tumour composed of mature adipocytes enclosed by a fibrous capsule and is exceedingly rare in the middle ear [19]. Although the imaging features of middle ear lipomas are not described in the literature, they should have the same imaging features as lipomas anywhere, with clear borders and homogeneous fat density/intensity. Hamartomas are tumour-like developmental anomalies resulting in a disorderly overgrowth of mature tissues that are native to the area where they occur and arrange abnormally; they may be derived from any one of the three germinal layers [20], without a capsule, and have ill-defined margins, which may be multifocal [21].

MET is mainly differentiated from dermoid in the teratoma families. Dermoid of middle ear was similar to teratoma, female sex and left ear are predominant. The tissues undergo mature differentiation without malignancy potential. Macroscopically, the dermoid is a well-circumscribed polypoid mass with a soft tissue pedicle [22]. Imaging demonstrates a well-circumscribed mass, with fat surrounding a central core of soft tissue corresponding to a fibrovascular stalk [23]. Teratoma density or signal is more heterogeneous, is a more soft tissue, less fat, it may have cyst, calcification, and even tooth [12]. In some cases, the dermoid is difficult to differentiate from a teratoma on CT and MRI.

### 4.1 Examination and Treatment Strategy

The recommended examination strategy is conventional CT and enhanced MRI plus DWI. CT can show the extent of the lesion and bone, including the ossicular chain, walls of the tympanum, ET, and internal carotid artery canal. Enhanced MRI is superior to enhanced CT and can provide more details of the teratomas, such as small amounts of fat and cysts, and a more accurate display of the relationship between the teratoma and the internal carotid artery and internal jugular vein; DWI is also recommended to demonstrate cholesteatoma.

Complete surgical excision is the definitive treatment for MET, ideally as early as possible, to potentially alleviate the patient’s otorrhea and prevent further extension.

## CONCLUSION

MET is an extremely rare condition that shows female sex and left ear predominance and is infrequently accompanied by ear malformation. The main symptoms are otorrhea and hearing loss. If an infant or child repeatedly presents with otorrhea that resists medical treatment, the patient should undergo CT and MRI examinations. Complete surgical excision is the definitive treatment.

## LIMITATIONS

There are some limitations to this study. This study was from a single hospital, and the number of cases was small, especially ear malformations with teratomas. Microtia with a false bony canal behind the EAC is extremely rare, and we plan to explore the clinical and imaging features of this rare malformation.

## Figures and Tables

**Table 1:** MET symptoms.

**No./Author,** **Year**	**Sex**	**Age**	**Otorrhea Age**	**Laterality**	**Otorrhea/** **Otitis ** **Media**	**Hearing Loss**	**Physical Examination**	**Special Medical History**
1	F	7 Y	1 Y	L	+	+	EAC and tympanum postoperative condition.	Tympanoplasty and partial excision of the MET five years ago.
2	F	2 Y	11 M	L	+	+	Tympanic membrane hyperaemia with granuloma.	With cholesteatoma in the tympnum.
3	F	20 Y	6 M	L	+	+	Amount of purulent secretion and granulation in left EAC.	-
4	F	7 Y	1 M	L	+	+	Full of granulation tissue in left EAC.	-
5	F	10 M	9 M	L	+	+	A mass covered with inflammatory granulation tissue in left EAC.	With cholesteatoma in the tympanum.
6	F	4 M	3 M	L	+	+	Purulent secretion and granulation in left EAC.	-
7	F	8Y	-	R	-	+	Right microtia, facial paralysis and EAC stenosis	MET was found in the plastic operation for microtia.
8	M	19 Y	-	R	-	+	Right microtia and EAC stenosis	MET was found in the plastic operation for microtia, and biopsy.
Silverstein H 1967 [5]	F	22Y	Birth	R	+	+	Facial paralysis, swelling behind the right ear.	Four times operation for remove the MET.
Forrest, AW 1993 [6]	F	8 M	2 M	L	+	NM	Polypoid granulation tissue on the posterosuperior canal wall.There was a yellowish mass medial to the anterior half of the tympanic membrane	After the first surgery, the remained teratoma in the ET slipped into pharynx and caused acute airway obstruction.
Navarro Cunchillos M 1996 [7]	M	2W	-	L	-	NM	Facial paralysis, a laterocervical mass.	-
Ruah Carlos B 1999 [4]	F	4 M	10 W	R	+	NM	Right EAC was filled with a soft mass embedded in pus.	-
Roncaroli F 2001 [8]	F	3Y	2 Y	R	+	+	Tympanic membrane to be under pressure and inflamed.	-
Wasinwong Y 2003 [9]	F	1W	-	L	-	+	Tongue-like mass in her mouth.	-
Min Chen 2011 [10]	F	10 M	6 M	L	+	NM	Anterior–inferior part of the tympanic membrane was bulging with a mass, a white mass in the nasopharynx.	Nasal obstruction, obstructive sleep apnoea.
Li JY 2022 [11]	M	48Y	Infant	L	+	+	A large amount of pus in the left EAC, a fleshy polyp present at a deeper site.	Underwent a surgery at another hospital.
Bowyer DJ 2012 [12]	F	10Y	Infant	L	+	+	A fleshy polyp arising from the middle ear extending through a perforation in the tympanic membrane into the EAC.	-
Alqurashi A 2015 [13]	F	3.5 M	3.5 M	L	+	NM	A protruded mass from theEAC.	-
Cruz-Toro P 2015 [14]	F	42Y	Infant	R	+	NM	NM	At the age of 6, had a unsuccessful mastoidectomy.
León F 2015 [15]	F	18Y	-	L	-	+	A noninflammatory swelling of the posterior wall of the EAC.	-
Wang J 2016 [16]	F	10 M	NM	L	+	+	A white mass obscuring the entire tympanic membrane.	With cholesteatoma in the tympnum.
Rondenet C 2017 [3]	F	3 Y	NM	L	+	NM	A mass effect in the left portion of the nasopharynx.	-

**Abbreviations:** MET middle ear teratoma, EAC external auditory canal, ET eustachian tube, F female, M male, L left, R right, Y year, M month, W week, NM not mentioned.

**Table 2:** The imaging features of MET.

**No./Author,** **Year**	**Location**	**Fat**	**Tooth/** **Calcification**	**Capsule**	**Destruction**	**Enhancment**	**Diagnosis**
1	Bony ET, parapharyngeal space.	+	+	+	ET expanded. Anteroinferior bony wall of the tympanum	Capsule Enhanced	Otitis media, by CT. Teratoma, by MRI.
2	Bony ET, parapharyngeal space, middle-inferior tympanum.	+	-	+	ET expanded, and anteroinferior bony wall of the tympanum was absorbed.	Plain scan	Benign mass, by CT; Teratoma, by MRI.
3	EAC, tympanum, ET, internal carotid artery canal, parapharyngeal space.	+	-	+ Sandwich sign	ET, middle-inferior tympanium and EAC expanded, anteroinferior wall of the tympanum destruction, the internal carotid artery canal slight destruction.	Central soft and capsule enhanced.	Teratoma, by CT and MRI.
4	EAC, tympanum, ET.	+	-	+ Sandwich sign	ET and middle-inferior tympanium expanded, anteroinferior wall of the tympanum was destroyed.	Soft tissue and capsule enhanced.	Cholesteatoma, by CT and MRI
5	EAC, tympanum, ET, parapharyngeal space.	+	-	+ Sandwich sign	-	Plain scan	Dermoid, by MRI
6	EAC, tympanum, antrum, ET,	+	-	+	ET,tympnum,EAC expanded	Plain scan	Otitis media, by CT
7	Tympanum, back of EAC, posterior styloid space.	+	-	+	The floor of the tympanic cavity was the destruction	Plain scan	Mass, by CT
8	Tympanum, back of EAC, anterior styloid and parapharyngeal spaces.	+	-	+ Sandwich sign	The floor of the tympanic cavity was destruction	Soft tissue enhanced.	Cholesteatoma, by CT, MRI
Silverstein H, 1967 [5]	EAC, postaural area, tympanum, ET, petrous apex, jugular bulb.	-	-	-	-	-	-
Forrest, A. W., 1993 [6]	Mastoid antrum, tympanum, ET, nasopharynx. Be attached medially to the internal carotid artery.	NM	NM	NM	ET was splayed.	NM	Congenital cholesteatoma.
Navarro Cunchillos, M, 1996 [7]	Middle ear cavity, ET, lateral cervical space.	-	+	NM	NM	NM	Tuberculosis or a congenital cholesteatoma
Ruah, Carlos B, 1999 [4]	ET, middle ear, EAC.	NM	NM	NM	NM	NM	NM
Roncaroli F, 2001 [8]	Middle ear.	NM	-	NM	Septal air cells	NM	Cholesteatoma
Wasinwong Y, 2003 [9]	ET, hypopharynx.	+	NM	NM	NM	Nonenhanced	NM
Min Chen, 2011 [10]	ET, nasopharynx, hypotympanum.	+	NM	+	Widen the left ET	A margin-enhancing core-like structure.	NM
Li JY, 2022 [11]	Tympanum, EAC, ET,	+	NM	+	NM	Capsule was enhanced slightly.	NM
Bowyer, D. J., 2012 [12]	ET, tympnum, EAC, infratemporal.	+	+	+	NM	Capsule was enhanced.	A germ cell tumour.
Alqurashi A., 2015 [13]	Tympanum, mastoid, EAC.	-	-	NM	NM	Minimal homogeneous enhancement	NM
Cruz-Toro, P., 2015 [14]	ET.	-	A molar in the Eustachian tube.	NM	Widen the ET	NM	Mature teratoma
León, F., 2015 [15]	Middle ear and mastoid, EAC, subcutaneous tissue surrounding the EAC.	NM	NM	NM	A small bone dehiscence in the tegmen tympani.	NM	NM
Wang, J., 2016 [16]	Middle ear, ET and mastoid cavity.	NM	NM	NM	NM	NM	Cholesteatoma
Rondenet, C., 2017 [3]	ET, nasopharynx.	+	-	NM	NM	Nonenhanced.	NM

**Abbreviations:** MET middle ear teratoma, EAC external auditory canal, ET eustachian tube, NM not mentioned.

## Data Availability

Not applicable.

## LIST OF ABBREVIATIONS

EAC: External Auditory Canal
ET: Eustachian Tube
ME: Middle Ear
MET: Middle Ear Teratoma
